# 1,5-Dimethyl-4-{[(3-methyl-5-oxo-1-phenyl-4,5-dihydro-1*H*-pyrazol-4-yl­idene)(thio­phen-2-yl)meth­yl]amino}-2-phenyl-1*H*-pyrazol-3(2*H*)-one

**DOI:** 10.1107/S1600536811002467

**Published:** 2011-01-22

**Authors:** Hualing Zhu, Litong Ban, Pingping Zhang, Xinxin Zhao, Junjie Ren

**Affiliations:** a Department of Basic Science, Tianjin Agricultural College, Tianjin Jinjing Road No. 22, Tianjin 300384, People’s Republic of China; b Department of Agricultural Science, Tianjin Agricultural College, Tianjin Jinjing Road No. 22, Tianjin 300384, People’s Republic of China; c Department of Food Science, Tianjin Agricultural College, Tianjin Jinjing Road No. 22, Tianjin 300384, People’s Republic of China

## Abstract

In the title compound, C_26_H_23_N_5_O_2_S, an intra­molecular N—H⋯O inter­action generates an *S*(6) ring. The essentially planar *S*(6) and pyrazole rings [maximum deviations = −0.0270 (14) and 0.0195 (15) Å, respectively] are nearly coplanar, making a dihedral angle of 3.94 (6)°. The *S*(6) ring makes dihedral angles of 23.79 (6), 78.53 (6) and 67.91 (6)° with the pyrazolone ring, the pyrazole ring and the benzene ring of anti­pyrine, respectively. The structure exhibits a thienyl-ring flip disorder with occupancy factors in the ratio 0.82:0.18.

## Related literature

For general background to pyrazolo­nes, see: Casas *et al.* (2007 ▶). For the anti­bacterial activity of pyrazolone Schiff bases, see: Zhang *et al.* (2008 ▶); Li *et al.* (2000 ▶). For our previous work in this area, see: Zhu *et al.* (2010*a* ▶,*b* ▶). For related structures, see: Shi *et al.* (2005 ▶); Goh *et al.* (2009 ▶). For disordered thienyl rings, see: Crundwell *et al.* (2003 ▶).
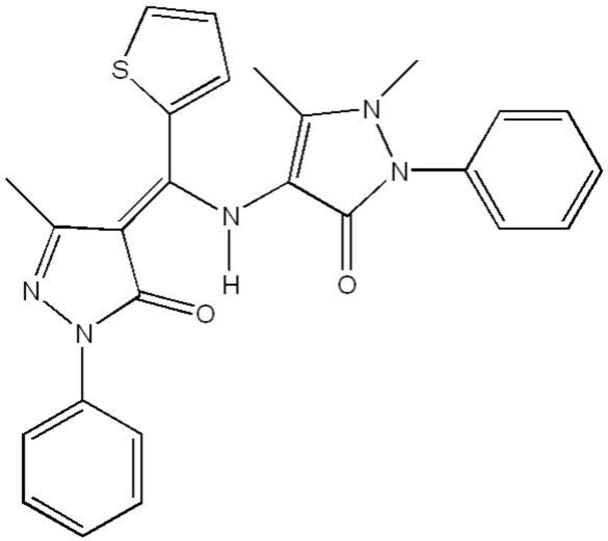


## Experimental

### 

#### Crystal data

C_26_H_23_N_5_O_2_S
*M* *_r_* = 469.55
Monoclinic, 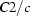

*a* = 27.098 (3) Å
*b* = 7.9045 (8) Å
*c* = 22.308 (2) Å
β = 99.011 (8)°
*V* = 4719.4 (9) Å^3^
*Z* = 8
Mo *K*α radiation
μ = 0.17 mm^−1^
*T* = 293 K
0.42 × 0.36 × 0.34 mm

#### Data collection

Rigaku Saturn diffractometer
Absorption correction: multi-scan (*CrystalClear*; Rigaku, 2008 ▶) *T*_min_ = 0.932, *T*_max_ = 0.944
22080 measured reflections
5570 independent reflections
3806 reflections with *I* > 2σ(*I*)
*R*_int_ = 0.037

#### Refinement

*R*[*F* ^2^ > 2σ(*F* ^2^)] = 0.049
*wR*(*F* ^2^) = 0.144
*S* = 1.02
5570 reflections
322 parameters
22 restraints
H-atom parameters constrained
Δρ_max_ = 0.23 e Å^−3^
Δρ_min_ = −0.25 e Å^−3^

### 

Data collection: *CrystalClear* (Rigaku, 2008 ▶); cell refinement: *CrystalClear*; data reduction: *CrystalClear*; program(s) used to solve structure: *SHELXS97* (Sheldrick, 2008 ▶); program(s) used to refine structure: *SHELXL97* (Sheldrick, 2008 ▶); molecular graphics: *ORTEPIII* (Burnett & Johnson, 1996 ▶) and *ORTEP-3 for Windows* (Farrugia, 1997 ▶); software used to prepare material for publication: *SHELXL97*.

## Supplementary Material

Crystal structure: contains datablocks I, global. DOI: 10.1107/S1600536811002467/dn2653sup1.cif

Structure factors: contains datablocks I. DOI: 10.1107/S1600536811002467/dn2653Isup2.hkl

Additional supplementary materials:  crystallographic information; 3D view; checkCIF report

## Figures and Tables

**Table 1:** Hydrogen-bond geometry (Å, °)

*D*—H⋯*A*	*D*—H	H⋯*A*	*D*⋯*A*	*D*—H⋯*A*
N3—H3⋯O2	0.86	1.96	2.6631 (18)	138

**Fig. 1.**
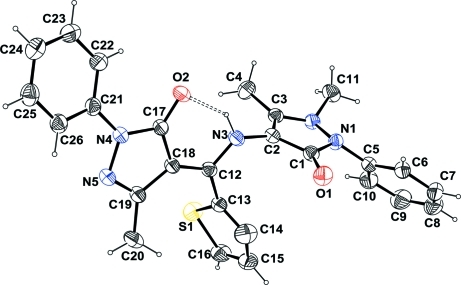
The molecular structure of the title compound with the atom numbering scheme. Displacement ellipsoids are drawn at the 30% probability level. H atoms are presented as a small spheres of arbitrary radii. Only the major component of the disordered thienyl ring is represented for the sake of clarity.

## supplementary crystallographic information

### Comment

Pyrazolones form a very important class of heterocycles due to their properties and applications (Casas *et al.*, 2007). Schiff-bases derived from 1-phenyl-3-methyl-4-acyl-5-pyrazolone have found extensive application in coordination chemistry (Shi *et al.*, 2005)and in antibacterial activation (Zhang *et al.*, 2008; Li *et al.*, 2000). In continuation of our studies on pyrazolone schiff bases (Zhu *et al.*, 2010*a*,*b*), we herein report the crystal structure of the title pyrazole compound.

The molecular structure of the title compound is shown in Fig. 1. An intramolecular N—H···O interaction generates a six- membered ring, producing an S(6) ring (O2 N3 C12 C17 C18), which stablizing the enamine–keto form of the compound. The S(6) ring and pyrazole ring (N4 N5 C17 C18 C19) are essentially planar,with the maximum deviations of -0.0270 (14) and 0.0195 (15) Å, respectively, at atoms C12 and C17.The two rings are coplanar to one another, as indicated by the dihedral angle formed between them of 3.94 (6)°. The S(6) ring makes dihedral angles of 23.79 (6)°,78.53 (6)° and 67.91 (6)° with the benzene ring of pyrazolone, the pyrazole ring and benzene ring of antipyrine,respectively. The bond lengths and angles agree well with those closely related pyrazole structures (Goh *et al.*, 2009)

The structure exhibits a thienyl-ring flip disorder with the occupancy factors in the ratio 82/18.

### Experimental

The title compound was synthesized by refluxing the mixture of 1-phenyl-3-methyl-4-(2-thenoyl)pyrazolone-5 (HPMTP) (15*m* mol) and 4-antipyrine (15*m* mol) in ethanol (100 ml) over a steam bath for about 4 h, then the solution was cooled down to room temperature. After seven days, pale yellow block was obtained and dried in air. The product was recrystallized from ethanol which afforded pale yellow and acerate crystals suitable for *X*–ray analysis.

### Refinement

During refinement, the thienyl ring showed evidence of ring-flip disorder which is common for unsubstituted 2- and 3-thienyl rings (Crundwell *et al.*, 2003). After finding three of the flipped disordered atoms in the difference map, the rest of the ring was generated and modeled. The occupancy factors of the disordered thienyl ring were first refine restraining the sum of the occupancy factore to be equal to 1.0. Once stabilised, the occupancy factors were fixed and not refined anymore. The final model suggested that the thienyl ring disorder was in the ratio 82/18. The disordered model was refined using the tools available in SHELXL-97 (Sheldrick, 2008): SADI for restraining distances, FLAT for constraining the thienyl rings to be planar, EXYZ for linking atoms occupying the same site and EADP to correlate anisotropic thermal parameters for related disordered atoms.

All H atoms were geometrically positioned and treated as riding on their parent atoms, with C—H = 0.93 Å for the aromatic, 0.96 Å for the methyl and N-H= 0.86 Å with *U*_iso_(H)= 1.2 *U*_eq_(Caromatic, N) or, 1.5*U*_eq_(Cmethyl).

### Crystal data

**Table:**

C_26_H_23_N_5_O_2_S	*F*(000) = 1968
*M**_r_* = 469.55	*D*_x_ = 1.322 Mg m^−^^3^
Monoclinic, *C*2/*c*	Mo *K*α radiation, λ = 0.71075 Å
Hall symbol: -C 2yc	Cell parameters from 5913 reflections
*a* = 27.098 (3) Å	θ = 2.6–27.9°
*b* = 7.9045 (8) Å	µ = 0.17 mm^−^^1^
*c* = 22.308 (2) Å	*T* = 293 K
β = 99.011 (8)°	Prism, colourless
*V* = 4719.4 (9) Å^3^	0.42 × 0.36 × 0.34 mm
*Z* = 8	

### Data collection

**Table:**

Rigaku Saturn diffractometer	5570 independent reflections
Radiation source: rotating anode	3806 reflections with *I* > 2σ(*I*)
multilayer	*R*_int_ = 0.037
Detector resolution: 7.31 pixels mm^-1^	θ_max_ = 27.9°, θ_min_ = 2.6°
ω scans	*h* = −35→35
Absorption correction: multi-scan (*CrystalClear*; Rigaku, 2008)	*k* = −10→8
*T*_min_ = 0.932, *T*_max_ = 0.944	*l* = −29→29
22080 measured reflections	

### Refinement

**Table:**

Refinement on *F*^2^	Primary atom site location: structure-invariant direct methods
Least-squares matrix: full	Secondary atom site location: difference Fourier map
*R*[*F*^2^ > 2σ(*F*^2^)] = 0.049	Hydrogen site location: inferred from neighbouring sites
*wR*(*F*^2^) = 0.144	H-atom parameters constrained
*S* = 1.02	*w* = 1/[σ^2^(*F*_o_^2^) + (0.0825*P*)^2^] where *P* = (*F*_o_^2^ + 2*F*_c_^2^)/3
5570 reflections	(Δ/σ)_max_ = 0.003
322 parameters	Δρ_max_ = 0.23 e Å^−^^3^
22 restraints	Δρ_min_ = −0.25 e Å^−^^3^

### Special details

**Table:**

Geometry. All esds (except the esd in the dihedral angle between two l.s. planes) are estimated using the full covariance matrix. The cell esds are taken into account individually in the estimation of esds in distances, angles and torsion angles; correlations between esds in cell parameters are only used when they are defined by crystal symmetry. An approximate (isotropic) treatment of cell esds is used for estimating esds involving l.s. planes.
Refinement. Refinement of *F*^2^ against ALL reflections. The weighted *R*-factor *wR* and goodness of fit *S* are based on *F*^2^, conventional *R*-factors *R* are based on *F*, with *F* set to zero for negative *F*^2^. The threshold expression of *F*^2^ > σ(*F*^2^) is used only for calculating *R*-factors(gt) *etc*. and is not relevant to the choice of reflections for refinement. *R*-factors based on *F*^2^ are statistically about twice as large as those based on *F*, and *R*- factors based on ALL data will be even larger.

### Fractional atomic coordinates and isotropic or equivalent isotropic displacement parameters (Å^2^)

**Table:**

	*x*	*y*	*z*	*U*_iso_*/*U*_eq_	Occ. (<1)
O1	0.25433 (4)	1.02510 (13)	0.08661 (6)	0.0607 (3)	
O2	0.08792 (4)	0.72515 (16)	−0.01432 (5)	0.0574 (3)	
N1	0.30507 (4)	0.78662 (15)	0.09168 (6)	0.0454 (3)	
N2	0.29718 (5)	0.61104 (15)	0.09307 (6)	0.0453 (3)	
N3	0.17055 (5)	0.75910 (17)	0.06681 (6)	0.0490 (3)	
H3	0.1558	0.7573	0.0298	0.059*	
N4	0.01707 (5)	0.77052 (16)	0.03130 (6)	0.0483 (3)	
N5	0.00673 (5)	0.81903 (18)	0.08851 (7)	0.0531 (4)	
C1	0.25902 (6)	0.87031 (19)	0.08467 (7)	0.0440 (4)	
C2	0.22290 (5)	0.73667 (18)	0.07704 (7)	0.0418 (3)	
C3	0.24689 (6)	0.58580 (18)	0.07935 (7)	0.0431 (3)	
C4	0.22546 (7)	0.4148 (2)	0.06696 (9)	0.0652 (5)	
H4A	0.1900	0.4190	0.0665	0.098*	
H4B	0.2402	0.3383	0.0981	0.098*	
H4C	0.2323	0.3762	0.0283	0.098*	
C5	0.35010 (6)	0.8549 (2)	0.12487 (7)	0.0473 (4)	
C6	0.36892 (6)	1.0029 (2)	0.10435 (8)	0.0560 (4)	
H6	0.3523	1.0563	0.0698	0.067*	
C7	0.41249 (7)	1.0712 (3)	0.13541 (11)	0.0717 (6)	
H7	0.4250	1.1720	0.1223	0.086*	
C8	0.43721 (8)	0.9902 (3)	0.18544 (12)	0.0836 (7)	
H8	0.4667	1.0357	0.2060	0.100*	
C9	0.41887 (8)	0.8425 (3)	0.20554 (10)	0.0843 (6)	
H9	0.4363	0.7876	0.2392	0.101*	
C10	0.37464 (7)	0.7747 (3)	0.17596 (9)	0.0655 (5)	
H10	0.3616	0.6765	0.1903	0.079*	
C11	0.33387 (7)	0.4992 (2)	0.07232 (9)	0.0628 (5)	
H11A	0.3337	0.3918	0.0924	0.094*	
H11B	0.3665	0.5489	0.0816	0.094*	
H11C	0.3256	0.4832	0.0293	0.094*	
C12	0.14164 (6)	0.78314 (18)	0.11001 (7)	0.0431 (4)	
S1	0.16071 (3)	0.63681 (8)	0.22363 (3)	0.0702 (2)	0.82
C13	0.16636 (6)	0.79831 (19)	0.17339 (7)	0.0468 (4)	0.82
C14	0.19533 (11)	0.9188 (3)	0.20297 (12)	0.0704 (8)	0.82
H14	0.2031	1.0169	0.1834	0.085*	0.82
C15	0.21288 (18)	0.8917 (6)	0.26270 (14)	0.0765 (8)	0.82
H15	0.2330	0.9675	0.2874	0.092*	0.82
C16	0.19767 (14)	0.7430 (5)	0.28143 (13)	0.0709 (9)	0.82
H16	0.2060	0.7018	0.3208	0.085*	0.82
S1'	0.20046 (19)	0.9710 (5)	0.1965 (2)	0.0702 (2)	0.18
C13'	0.16636 (6)	0.79831 (19)	0.17339 (7)	0.0468 (4)	0.18
C14'	0.1637 (5)	0.6894 (16)	0.2181 (5)	0.0704 (8)	0.18
H14'	0.1463	0.5877	0.2123	0.085*	0.18
C15'	0.1879 (8)	0.738 (2)	0.2721 (6)	0.0765 (8)	0.18
H15'	0.1895	0.6745	0.3075	0.092*	0.18
C16'	0.2097 (7)	0.889 (2)	0.2692 (6)	0.0709 (9)	0.18
H16'	0.2278	0.9432	0.3025	0.085*	0.18
C17	0.06744 (5)	0.75638 (19)	0.03103 (7)	0.0449 (4)	
C18	0.09039 (6)	0.78841 (18)	0.09237 (7)	0.0437 (4)	
C19	0.04936 (6)	0.8289 (2)	0.12425 (8)	0.0504 (4)	
C20	0.05032 (7)	0.8851 (3)	0.18855 (9)	0.0727 (6)	
H20A	0.0181	0.9285	0.1934	0.109*	
H20B	0.0750	0.9721	0.1982	0.109*	
H20C	0.0585	0.7907	0.2153	0.109*	
C21	−0.02273 (6)	0.75001 (19)	−0.01723 (7)	0.0468 (4)	
C22	−0.01595 (7)	0.6573 (2)	−0.06807 (8)	0.0579 (4)	
H22	0.0149	0.6082	−0.0706	0.069*	
C23	−0.05544 (7)	0.6384 (2)	−0.11491 (9)	0.0648 (5)	
H23	−0.0511	0.5759	−0.1490	0.078*	
C24	−0.10124 (7)	0.7112 (2)	−0.11174 (9)	0.0650 (5)	
H24	−0.1276	0.6986	−0.1436	0.078*	
C25	−0.10744 (7)	0.8021 (2)	−0.06118 (9)	0.0618 (5)	
H25	−0.1383	0.8510	−0.0588	0.074*	
C26	−0.06879 (6)	0.8225 (2)	−0.01381 (9)	0.0540 (4)	
H26	−0.0735	0.8844	0.0203	0.065*	

### Atomic displacement parameters (Å^2^)

**Table:**

	*U*^11^	*U*^22^	*U*^33^	*U*^12^	*U*^13^	*U*^23^
O1	0.0648 (7)	0.0375 (6)	0.0805 (9)	0.0072 (5)	0.0132 (6)	0.0016 (5)
O2	0.0493 (6)	0.0790 (8)	0.0465 (7)	0.0004 (6)	0.0160 (5)	−0.0109 (6)
N1	0.0438 (7)	0.0364 (7)	0.0564 (8)	0.0001 (5)	0.0095 (6)	−0.0004 (5)
N2	0.0477 (8)	0.0353 (7)	0.0541 (8)	0.0042 (5)	0.0122 (6)	−0.0015 (5)
N3	0.0451 (7)	0.0643 (8)	0.0390 (7)	0.0024 (6)	0.0108 (6)	−0.0003 (6)
N4	0.0422 (7)	0.0559 (8)	0.0494 (8)	−0.0021 (6)	0.0152 (6)	−0.0073 (6)
N5	0.0494 (8)	0.0609 (9)	0.0531 (8)	−0.0029 (6)	0.0206 (7)	−0.0073 (6)
C1	0.0466 (9)	0.0403 (8)	0.0462 (9)	0.0041 (7)	0.0108 (7)	0.0004 (6)
C2	0.0427 (8)	0.0444 (8)	0.0395 (8)	0.0028 (6)	0.0100 (6)	0.0004 (6)
C3	0.0506 (9)	0.0411 (8)	0.0393 (8)	−0.0014 (7)	0.0122 (7)	0.0008 (6)
C4	0.0727 (12)	0.0466 (10)	0.0756 (13)	−0.0091 (9)	0.0088 (10)	−0.0026 (8)
C5	0.0449 (9)	0.0492 (9)	0.0496 (9)	−0.0004 (7)	0.0132 (7)	−0.0053 (7)
C6	0.0548 (10)	0.0545 (10)	0.0615 (11)	−0.0064 (8)	0.0180 (8)	−0.0054 (8)
C7	0.0580 (12)	0.0731 (13)	0.0879 (15)	−0.0165 (10)	0.0234 (11)	−0.0217 (11)
C8	0.0542 (12)	0.1040 (18)	0.0905 (17)	−0.0113 (12)	0.0047 (12)	−0.0319 (14)
C9	0.0697 (14)	0.1072 (18)	0.0688 (14)	0.0025 (13)	−0.0116 (11)	−0.0062 (13)
C10	0.0655 (12)	0.0736 (13)	0.0562 (11)	−0.0005 (10)	0.0059 (9)	0.0044 (9)
C11	0.0650 (11)	0.0491 (10)	0.0797 (13)	0.0140 (8)	0.0277 (10)	−0.0015 (9)
C12	0.0502 (9)	0.0384 (8)	0.0426 (8)	−0.0014 (6)	0.0134 (7)	0.0004 (6)
S1	0.0933 (5)	0.0628 (5)	0.0532 (4)	−0.0107 (4)	0.0077 (3)	0.0143 (3)
C13	0.0493 (9)	0.0510 (9)	0.0418 (8)	0.0006 (7)	0.0129 (7)	−0.0007 (7)
C14	0.111 (2)	0.0564 (18)	0.0461 (14)	−0.0152 (16)	0.0183 (13)	0.0058 (12)
C15	0.0871 (19)	0.096 (2)	0.0469 (15)	−0.0203 (15)	0.0134 (14)	−0.0138 (14)
C16	0.079 (2)	0.093 (2)	0.0389 (14)	0.0077 (16)	0.0025 (13)	0.0086 (14)
S1'	0.0933 (5)	0.0628 (5)	0.0532 (4)	−0.0107 (4)	0.0077 (3)	0.0143 (3)
C13'	0.0493 (9)	0.0510 (9)	0.0418 (8)	0.0006 (7)	0.0129 (7)	−0.0007 (7)
C14'	0.111 (2)	0.0564 (18)	0.0461 (14)	−0.0152 (16)	0.0183 (13)	0.0058 (12)
C15'	0.0871 (19)	0.096 (2)	0.0469 (15)	−0.0203 (15)	0.0134 (14)	−0.0138 (14)
C16'	0.079 (2)	0.093 (2)	0.0389 (14)	0.0077 (16)	0.0025 (13)	0.0086 (14)
C17	0.0441 (9)	0.0447 (8)	0.0481 (9)	−0.0024 (7)	0.0144 (7)	−0.0034 (6)
C18	0.0440 (9)	0.0450 (8)	0.0447 (8)	−0.0030 (6)	0.0149 (7)	−0.0023 (6)
C19	0.0507 (10)	0.0533 (9)	0.0509 (9)	−0.0034 (7)	0.0197 (8)	−0.0041 (7)
C20	0.0693 (12)	0.0975 (16)	0.0566 (11)	0.0001 (10)	0.0262 (9)	−0.0158 (10)
C21	0.0442 (9)	0.0437 (8)	0.0537 (9)	−0.0050 (7)	0.0113 (7)	0.0004 (7)
C22	0.0509 (10)	0.0610 (11)	0.0621 (11)	0.0011 (8)	0.0097 (8)	−0.0105 (8)
C23	0.0668 (12)	0.0650 (12)	0.0613 (12)	−0.0039 (9)	0.0056 (9)	−0.0120 (9)
C24	0.0592 (11)	0.0631 (12)	0.0688 (12)	−0.0045 (9)	−0.0019 (9)	0.0008 (9)
C25	0.0492 (10)	0.0544 (11)	0.0810 (14)	0.0022 (8)	0.0077 (9)	0.0043 (9)
C26	0.0488 (10)	0.0487 (9)	0.0660 (11)	−0.0009 (7)	0.0138 (8)	−0.0033 (8)

### Geometric parameters (Å, °)

**Table:**

O1—C1	1.2316 (17)	C12—C18	1.384 (2)
O2—C17	1.2524 (16)	C12—C13	1.472 (2)
N1—C1	1.3994 (19)	S1—C13	1.7213 (16)
N1—N2	1.4054 (17)	S1—C16	1.722 (3)
N1—C5	1.430 (2)	C13—C14	1.340 (3)
N2—C3	1.3637 (19)	C14—C15	1.360 (4)
N2—C11	1.4590 (19)	C14—H14	0.9300
N3—C12	1.3471 (18)	C15—C16	1.335 (3)
N3—C2	1.412 (2)	C15—H15	0.9300
N3—H3	0.8600	C16—H16	0.9300
N4—C17	1.3703 (19)	S1'—C16'	1.728 (10)
N4—N5	1.4023 (17)	C14'—C15'	1.336 (8)
N4—C21	1.413 (2)	C14'—H14'	0.9300
N5—C19	1.299 (2)	C15'—C16'	1.336 (8)
C1—C2	1.432 (2)	C15'—H15'	0.9300
C2—C3	1.355 (2)	C16'—H16'	0.9300
C3—C4	1.480 (2)	C17—C18	1.434 (2)
C4—H4A	0.9600	C18—C19	1.446 (2)
C4—H4B	0.9600	C19—C20	1.498 (2)
C4—H4C	0.9600	C20—H20A	0.9600
C5—C10	1.380 (2)	C20—H20B	0.9600
C5—C6	1.383 (2)	C20—H20C	0.9600
C6—C7	1.382 (3)	C21—C26	1.386 (2)
C6—H6	0.9300	C21—C22	1.387 (2)
C7—C8	1.368 (3)	C22—C23	1.382 (3)
C7—H7	0.9300	C22—H22	0.9300
C8—C9	1.371 (3)	C23—C24	1.380 (3)
C8—H8	0.9300	C23—H23	0.9300
C9—C10	1.382 (3)	C24—C25	1.370 (3)
C9—H9	0.9300	C24—H24	0.9300
C10—H10	0.9300	C25—C26	1.376 (3)
C11—H11A	0.9600	C25—H25	0.9300
C11—H11B	0.9600	C26—H26	0.9300
C11—H11C	0.9600		
			
C1—N1—N2	109.46 (11)	C18—C12—C13	123.81 (13)
C1—N1—C5	123.58 (12)	C13—S1—C16	91.47 (12)
N2—N1—C5	118.79 (12)	C14—C13—C12	132.40 (16)
C3—N2—N1	106.84 (11)	C14—C13—S1	108.14 (15)
C3—N2—C11	123.14 (13)	C12—C13—S1	119.45 (12)
N1—N2—C11	118.55 (12)	C13—C14—C15	117.3 (3)
C12—N3—C2	125.74 (14)	C13—C14—H14	121.3
C12—N3—H3	117.1	C15—C14—H14	121.3
C2—N3—H3	117.1	C16—C15—C14	111.6 (3)
C17—N4—N5	111.56 (13)	C16—C15—H15	124.2
C17—N4—C21	128.95 (13)	C14—C15—H15	124.2
N5—N4—C21	119.43 (12)	C15—C16—S1	111.5 (3)
C19—N5—N4	106.83 (12)	C15—C16—H16	124.2
O1—C1—N1	124.12 (14)	S1—C16—H16	124.2
O1—C1—C2	131.66 (14)	C15'—C14'—H14'	122.8
N1—C1—C2	104.21 (12)	C16'—C15'—C14'	111.7 (12)
C3—C2—N3	125.48 (14)	C16'—C15'—H15'	124.1
C3—C2—C1	109.25 (13)	C14'—C15'—H15'	124.1
N3—C2—C1	125.25 (13)	C15'—C16'—S1'	112.5 (11)
C2—C3—N2	109.66 (13)	C15'—C16'—H16'	123.7
C2—C3—C4	128.65 (15)	S1'—C16'—H16'	123.7
N2—C3—C4	121.65 (14)	O2—C17—N4	125.97 (15)
C3—C4—H4A	109.5	O2—C17—C18	128.66 (14)
C3—C4—H4B	109.5	N4—C17—C18	105.36 (12)
H4A—C4—H4B	109.5	C12—C18—C17	122.15 (13)
C3—C4—H4C	109.5	C12—C18—C19	132.96 (15)
H4A—C4—H4C	109.5	C17—C18—C19	104.85 (13)
H4B—C4—H4C	109.5	N5—C19—C18	111.29 (14)
C10—C5—C6	120.45 (17)	N5—C19—C20	119.07 (14)
C10—C5—N1	121.06 (15)	C18—C19—C20	129.57 (16)
C6—C5—N1	118.48 (15)	C19—C20—H20A	109.5
C7—C6—C5	119.67 (19)	C19—C20—H20B	109.5
C7—C6—H6	120.2	H20A—C20—H20B	109.5
C5—C6—H6	120.2	C19—C20—H20C	109.5
C8—C7—C6	119.8 (2)	H20A—C20—H20C	109.5
C8—C7—H7	120.1	H20B—C20—H20C	109.5
C6—C7—H7	120.1	C26—C21—C22	119.95 (16)
C7—C8—C9	120.6 (2)	C26—C21—N4	119.76 (15)
C7—C8—H8	119.7	C22—C21—N4	120.29 (14)
C9—C8—H8	119.7	C23—C22—C21	119.30 (16)
C8—C9—C10	120.4 (2)	C23—C22—H22	120.3
C8—C9—H9	119.8	C21—C22—H22	120.3
C10—C9—H9	119.8	C24—C23—C22	120.84 (18)
C5—C10—C9	119.1 (2)	C24—C23—H23	119.6
C5—C10—H10	120.5	C22—C23—H23	119.6
C9—C10—H10	120.5	C25—C24—C23	119.24 (18)
N2—C11—H11A	109.5	C25—C24—H24	120.4
N2—C11—H11B	109.5	C23—C24—H24	120.4
H11A—C11—H11B	109.5	C24—C25—C26	121.12 (17)
N2—C11—H11C	109.5	C24—C25—H25	119.4
H11A—C11—H11C	109.5	C26—C25—H25	119.4
H11B—C11—H11C	109.5	C25—C26—C21	119.55 (17)
N3—C12—C18	118.10 (14)	C25—C26—H26	120.2
N3—C12—C13	118.08 (14)	C21—C26—H26	120.2
			
C1—N1—N2—C3	7.51 (15)	N3—C12—C13—S1	−109.04 (14)
C5—N1—N2—C3	157.23 (12)	C18—C12—C13—S1	69.48 (18)
C1—N1—N2—C11	152.10 (15)	C16—S1—C13—C14	0.23 (16)
C5—N1—N2—C11	−58.19 (19)	C16—S1—C13—C12	179.22 (18)
C17—N4—N5—C19	−2.41 (18)	C12—C13—C14—C15	−179.3 (2)
C21—N4—N5—C19	−179.86 (14)	S1—C13—C14—C15	−0.51 (16)
N2—N1—C1—O1	174.18 (15)	C13—C14—C15—C16	0.6 (2)
C5—N1—C1—O1	26.2 (2)	C14—C15—C16—S1	−0.4 (2)
N2—N1—C1—C2	−4.47 (15)	C13—S1—C16—C15	0.1 (2)
C5—N1—C1—C2	−152.43 (14)	C14'—C15'—C16'—S1'	−1.0 (5)
C12—N3—C2—C3	97.57 (19)	N5—N4—C17—O2	−175.43 (15)
C12—N3—C2—C1	−84.5 (2)	C21—N4—C17—O2	1.7 (3)
O1—C1—C2—C3	−178.69 (17)	N5—N4—C17—C18	3.33 (16)
N1—C1—C2—C3	−0.18 (16)	C21—N4—C17—C18	−179.53 (14)
O1—C1—C2—N3	3.1 (3)	N3—C12—C18—C17	4.9 (2)
N1—C1—C2—N3	−178.36 (13)	C13—C12—C18—C17	−173.64 (14)
N3—C2—C3—N2	−176.91 (13)	N3—C12—C18—C19	−172.35 (16)
C1—C2—C3—N2	4.92 (17)	C13—C12—C18—C19	9.1 (3)
N3—C2—C3—C4	5.7 (3)	O2—C17—C18—C12	−2.1 (3)
C1—C2—C3—C4	−172.46 (16)	N4—C17—C18—C12	179.21 (14)
N1—N2—C3—C2	−7.60 (16)	O2—C17—C18—C19	175.83 (16)
C11—N2—C3—C2	−150.16 (14)	N4—C17—C18—C19	−2.88 (16)
N1—N2—C3—C4	170.00 (14)	N4—N5—C19—C18	0.41 (18)
C11—N2—C3—C4	27.4 (2)	N4—N5—C19—C20	177.69 (15)
C1—N1—C5—C10	117.51 (17)	C12—C18—C19—N5	179.15 (16)
N2—N1—C5—C10	−27.7 (2)	C17—C18—C19—N5	1.57 (18)
C1—N1—C5—C6	−63.0 (2)	C12—C18—C19—C20	2.2 (3)
N2—N1—C5—C6	151.78 (13)	C17—C18—C19—C20	−175.35 (18)
C10—C5—C6—C7	−0.2 (2)	C17—N4—C21—C26	−158.43 (15)
N1—C5—C6—C7	−179.70 (14)	N5—N4—C21—C26	18.5 (2)
C5—C6—C7—C8	1.3 (3)	C17—N4—C21—C22	21.8 (2)
C6—C7—C8—C9	−0.7 (3)	N5—N4—C21—C22	−161.24 (15)
C7—C8—C9—C10	−1.0 (3)	C26—C21—C22—C23	0.1 (3)
C6—C5—C10—C9	−1.4 (3)	N4—C21—C22—C23	179.81 (15)
N1—C5—C10—C9	178.03 (16)	C21—C22—C23—C24	0.3 (3)
C8—C9—C10—C5	2.0 (3)	C22—C23—C24—C25	−0.4 (3)
C2—N3—C12—C18	−174.54 (14)	C23—C24—C25—C26	0.2 (3)
C2—N3—C12—C13	4.1 (2)	C24—C25—C26—C21	0.1 (3)
N3—C12—C13—C14	69.7 (2)	C22—C21—C26—C25	−0.2 (2)
C18—C12—C13—C14	−111.8 (2)	N4—C21—C26—C25	−179.99 (15)

### Hydrogen-bond geometry (Å, °)

**Table:**

*D*—H···*A*	*D*—H	H···*A*	*D*···*A*	*D*—H···*A*
N3—H3···O2	0.86	1.96	2.6631 (18)	138
